# Perioperative serum syndecan-1 concentrations in patients who underwent cardiovascular surgery with cardiopulmonary bypass and its association with the occurrence of postoperative acute kidney injury: a retrospective observational study

**DOI:** 10.1186/s12871-024-02546-1

**Published:** 2024-04-22

**Authors:** Atsushi Miyazaki, Mai Hokka, Norihiko Obata, Satoshi Mizobuchi

**Affiliations:** Division of Anesthesiology, Department of Surgery Related, Kobe University Graduate School of Medicine, 7-5-2 Kusunoki Cho, Chuo-Ku, Kobe, Hyogo 650-0017 Japan

**Keywords:** Syndecan-1, Endothelial injury, Acute kidney injury

## Abstract

**Background:**

Various factors can cause vascular endothelial damage during cardiovascular surgery (CVS) with cardiopulmonary bypass (CPB), which has been suggested to be associated with postoperative complications. However, few studies have specifically investigated the relationship between the degree of vascular endothelial damage and postoperative acute kidney injury (pAKI).

The objectives of this study were to measure perioperative serum syndecan-1 concentrations in patients who underwent CVS with CPB, evaluate their trends, and determine their association with pAKI.

**Methods:**

This was a descriptive and case‒control study conducted at the National University Hospital. Adult patients who underwent CVS with CPB at a national university hospital between March 15, 2016, and August 31, 2020, were included. Patients who were undergoing preoperative dialysis, had preoperative serum creatinine concentrations greater than 2.0 mg dl^−1^, who were undergoing surgery involving the descending aorta were excluded. The perioperative serum syndecan-1 concentration was measured, and its association with pAKI was investigated.

**Results:**

Fifty-two patients were included. pAKI occurred in 18 (34.6%) of those patients. The serum syndecan-1 concentration increased after CPB initiation and exhibited bimodal peak values. The serum syndecan-1 concentration at all time points was significantly elevated compared to that after the induction of anesthesia. The serum syndecan-1 concentration at 30 min after weaning from CPB and on postoperative day 1 was associated with the occurrence of pAKI (OR = 1.10 [1.01 to 1.21],* P* = 0.03]; OR = 1.16 [1.01 to 1.34], *P* = 0.04]; and the cutoff values of the serum syndecan-1 concentration that resulted in pAKI were 101.0 ng ml^−1^ (sensitivity = 0.71, specificity = 0.62, area under the curve (AUC) = 0.67 (0.51 to 0.83)) and 57.1 ng ml^−1^ (sensitivity = 0.82, specificity = 0.56, AUC = 0.71 (0.57 to 0.86)). Multivariate logistic regression analysis revealed that the serum syndecan-1 concentration on postoperative day 1 was associated with the occurrence of pAKI (OR = 1.02 [1.00 to 1.03]; *P* = 0.03).

**Conclusion:**

The serum syndecan-1 concentration at all time points was significantly greater than that after the induction of anesthesia. The serum syndecan-1 concentration on postoperative day 1 was significantly associated with the occurrence of pAKI.

**Trial registration:**

This study is not a clinical trial and is not registered with the registry.

## Background

Various postoperative complications can lead to increased mortality in patients receiving cardiovascular surgery (CVS) with cardiopulmonary bypass (CPB). Postoperative AKI (pAKI) is an important complication that occurs in 5–30% of patients undergoing CVS, and even small increases in serum creatinine concentrations have been reported to lead to increased mortality [1]. Recently, it has been suggested that management with goal-directed perfusion focusing on oxygen supply during CPB period may reduce the incidence of pAKI, but pAKI is multifactorial [2] and there is no established prevention protocol. Vascular endothelial damage is thought to be one of the causes of pAKI [3–5]. Rehm et al. reported that the vascular endothelium in patients undergoing CVS is easily destroyed due to inflammation induced by CPB, aortic occlusion, and ischemia–reperfusion injury [6]. In addition, it has been reported that the vascular endothelial glycocalyx, which is present on the surface of vascular endothelial cells, is fragile and sheds when subjected to invasive stresses such as CVS, leading to increased plasma concentrations of vascular endothelial glycocalyx components such as syndecan-1 [7]. Although previous studies have investigated the association between preoperative or postoperative serum syndecan-1 concentrations and pAKI, few studies have examined perioperative serum syndecan-1 levels and investigated the association with pAKI. In the present study, we hypothesized that the perioperative serum syndecan-1 concentration is associated with pAKI. The objectives of this study were to examine the perioperative trends in the serum syndecan-1 concentration in adult patients receiving CVS via CPB and to clarify the association between vascular endothelial damage and the occurrence of pAKI.

## Methods

### Design

This was a single-center, retrospective, descriptive study performed to investigate the perioperative serum syndecan-1 concentration in patients who underwent CVS with CPB. This study was conducted using samples stored in a previous study [8].

Ethical approval for this study was provided by the Kobe University Graduate School of Medicine, Medical Ethics Committee (Chairperson Prof. Makoto Nakamura, approval number: B220044) on June 21, 2022.

A trained researcher collected the data and entered the information into a database. Data monitoring and source-data verification were conducted in accordance with a predefined plan. Due to the retrospective nature of the study, the need for informed consent was waived by the ethical committee. Instead, the researchers made appropriate disclosures about the study and provided the participants with the opportunity to refuse enrollment in the study.

### Setting and participants

Adult patients who underwent CVS with CPB at a national university hospital between March 15, 2016, and August 31, 2020, were included in this study. Patients who were undergoing preoperative dialysis, had preoperative serum creatinine concentrations greater than 2.0 mg dl^−1^, or who were undergoing surgery involving the descending aorta were excluded.

### Patient characteristics

The following patient characteristics were obtained: age, sex, weight, height, ASA-PS, European System for Cardiac Operative Risk Evaluation II, presence of hypertension and diabetes mellitus, preoperative left ventricular ejection fraction (LVEF), and estimated glomerular filtration rate (eGFR). Surgical information, including operation time, aortic cross-clamp time, duration of CPB, type of procedure, and amount of transfusion during surgery, was also obtained.

### Measurements of the serum syndecan-1 concentration

**Fig. 1:**
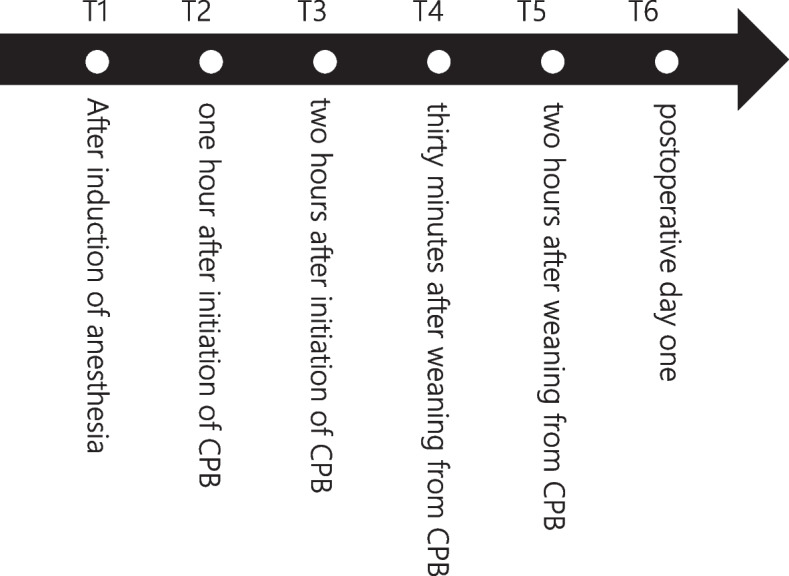
Serum syndecan-1 concentration was measured at each time point. The serum syndecan-1 concentration was measured at the following 6 time points: T1) after anesthesia induction, T2) 1 h after initiation of CPB, T3) 2 h after initiation of CPB, T4) 30 min after weaning from CPB, T5) 2 h after weaning from CPB, and T6) on postoperative day 1

Serum syndecan-1 concentrations were measured at the following six time points: (T1) after induction of anesthesia, (T2) 1 h after initiation of CPB, (T3) 2 h after initiation of CPB, (T4) 30 min after weaning from CPB, (T5) 2 h after weaning from CPB, and (T6) postoperative day 1 (Fig. 1). The serum syndecan-1 concentration was measured using an enzyme-linked immunosorbent assay (ELISA) kit (Human CD138 ELISA Kit, Diaclone SAS, France). To prevent observer bias, the serum syndecan-1 concentration was measured by a third party not involved in the study.

### Anesthesia methods

Anesthesia was administered by the anesthesiologist in charge. At the facility where this study was conducted, 500 mg of methylprednisolone was administered before starting CPB. Heparin was used as an anticoagulant to control the activated clotting time for more than 400 s, and protamine was administered as a heparin antagonist when hemostasis was confirmed after weaning from CPB. The choice of the type and amount of transfusion and inotropic agent also depended on the anesthesiologist in charge.

### CPB management

Several trained perfusionists controlled the circulatory dynamics during CPB period.

During the CPB procedure, the cardiac index was managed with the target of 2.4–2.6 L/min/m^2^, and oxygen delivery (DO_2_) and oxygen consumption (VO_2_) were monitored as needed.

### Study outcomes

The primary outcome was the change in the perioperative serum syndecan-1 concentration. The secondary outcome was the association between the perioperative serum syndecan-1 concentration and pAKI incidence. The diagnosis of pAKI was based on the Kidney Disease Improving Global Outcomes classification system. Although AKI was defined as an increase in the serum creatinine concentration and a decrease in urine output, the authors decided not to adopt the criterion of urine output for the definition of AKI in the present study to avoid the influence of the use of a diuretic drug in patients.

### Statistical analysis

All variables are expressed as medians 〔IQRs〕 or means ± SDs. First, the perioperative serum syndecan-1 concentrations were graphically plotted for all patients, and the serum syndecan-1 concentration at each measurement point were compared to that at T1. Comparisons between each measurement point were performed using the Mann‒Whitney U test. The patients were then divided into groups according to the presence or absence of pAKI, and the perioperative serum syndecan-1 concentration was graphically plotted for each group and compared at each time point. Comparisons between two groups were performed using the Mann‒Whitney U test. If the CPB duration was less than 2 h, the serum syndecan-1 concentration at T3 was not measured, and statistical analysis was performed only for those who had data.

A single regression analysis was performed to examine the association between serum syndecan-1 (syndecan-1/10) concentrations and pAKI at each time point. At the time points at which significant differences were detected in the single regression analysis, receiver operating characteristic (ROC) curve analysis was performed to estimate the cutoff value.

The χ2 test was used to analyze whether a serum syndecan-1 concentration above or below the cutoff value had an effect on the occurrence of pAKI.

Finally, multivariate logistic regression analysis was performed to evaluate the association between the serum syndecan-1 concentration and pAKI incidence. No statistical analysis for sample size determination was performed in this study, and a *P* value < 0.05 was considered to indicate a statistically significant difference. Statistical analysis was performed using SigmaPlot 14.5 (SYSTAT software, CA, United States).

## Results

Fifty-two patients who underwent CVS with CPB were included. pAKI occurred in 18 (34.6%) of those patients. Stage 1 and Stage 2 were 15 and 3, respectively.

### Patient demographic characteristics

Table 1 shows a comparison of the demographic data of the patients with and without pAKI. There were more male patients with pAKI than female patients (*P* = 0.01), and patients with AKI had a lower preoperative eGFR (*P* = 0.02) than did those without pAKI. There were no significant differences between the two groups with respect to the other demographic parameters.

**Table 1:** Characteristics of patients with and without postoperative AKI

	with pAKI (*n* = 18)	without pAKI (*n* = 34)	*P* value
age (years)	72〔63 to 78〕	74〔68 to 82〕	0.33
male, *n* (%)	16 (88.9)	17(50)	0.01
height (cm)	159.8 ± 11.4	161.8 ± 8.8	0.51
weight (kg)	63.5〔56.1 to 69.8〕	53.6〔47.8 to 66.3〕	0.07
ASA-PS	3〔2 to 3〕	3〔2 to 3〕	0.69
EuroSCORE II	3.55〔1.68 to 5.15〕	3.00〔2.04 to 4.61〕	0.33
hypertension, *n* (%)	11(61.1)	21(61.8)	1.00
diabetes mellitus, *n* (%)	5(27.8)	3(8.8)	0.11
preoperative eGFR (ml min^−1^ 1.73m^2−1^)	57.5 ± 13.0	67.5 ± 14.9	0.02
LVEF(%)	60.2 ± 13.5	62.4 ± 9.5	0.50
operation time (min)	290〔259 to 409〕	328〔266 to 403〕	0.57
aortic cross-clamp time (min)	112〔89 to 166〕	116〔90 to 147〕	0.97
duration of CPB (min)	166〔140 to 247〕	182〔134 to 222〕	0.86
transfusion (RCC) (unit)	6〔4 to 10]	6(0 to 11)	0.44
transfusion (FFP) (unit)	8(4 to 10)	8〔4 to 8〕	0.72
type of procedure, n. (%)			0.39
total arch replacement	4(22.2)	9(26.5)	
valve surgery	13(72.2)	19(55.9)	
total arch replacement + valve surgery	1(5.6)	6(17.6)	

*pAKI* postoperative acute kidney injury, *EuroSCOREII* European System for Cardiac Operative Risk Evaluation II, *eGFR* estimated glomerular filtration rate, *LVEF* left ventricular ejection fraction, *RCC* red cell concentrate, *FFP* fresh frozen plasma

### Changes in the serum syndecan-1 concentration

**Fig. 2:**
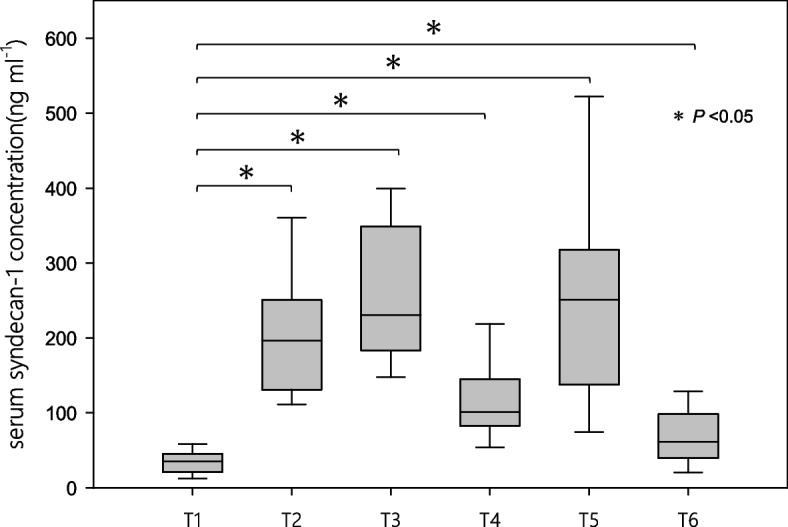
Perioperative serum syndecan-1 concentration. Shows the perioperative serum syndecan-1 concentrations at each time point. Serum syndecan-1 concentrations at all time points were significantly elevated compared to those at T1. * *P* < 0.05

Figure 2 shows the perioperative changes in the serum syndecan-1 concentration. Six patients with a CPB duration of less than 2 h did not have a serum syndecan-1 measurement at T3. It began to increase with the initiation of CPB and then reached a bimodal peak value. The serum syndecan-1 concentration at all time points was significantly elevated compared to that at T1.

### Comparison of the serum syndecan-1 concentration between patients with and without pAKI

**Fig. 3:**
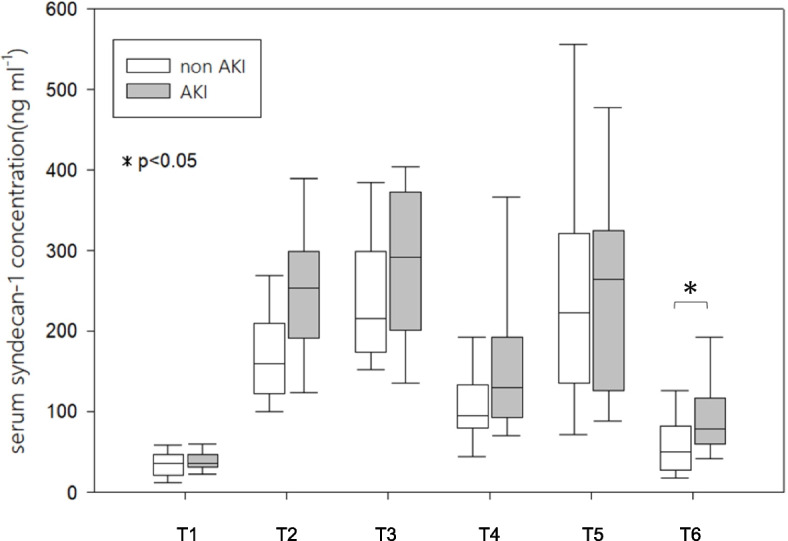
Comparisons of perioperative serum syndecan-1 concentrations in patients with and without pAKI. Shows a comparison of the perioperative serum syndecan-1 concentrations between patients with and without pAKI at the 6 time points. The white box plots indicate values for patients without pAKI, and the black box plots indicate values for patients with pAKI. * *P* < 0.05

**Fig. 4:**
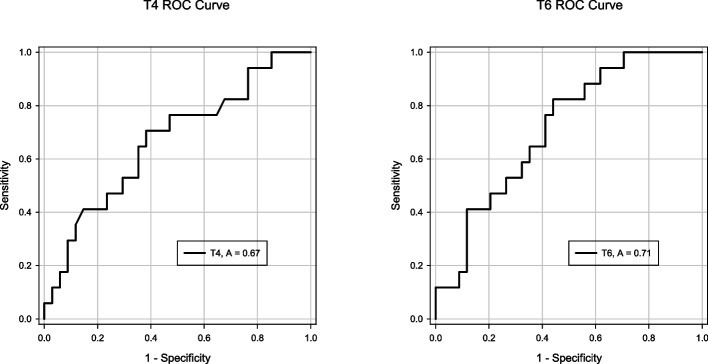
ROC curves of the serum syndecan-1 concentration at T4 and T6 for the prediction of pAKI. The black line represents the ROC curve for T4, and the red line represents that for T6

Figure 3 shows a comparison of perioperative serum syndecan-1 concentrations between patients with and without pAKI. Before and after weaning from CPB, the serum syndecan-1 concentration was not significantly different between the two groups, whereas there was a significant difference at T6 (patients with pAKI 97.1 〔73.5 to 127.2〕 ng ml^−1^ and patients without pAKI 53.3 〔38.7 to 74.4〕 ng ml^−1^ (*P* < 0.01)). Table 2 shows the ORs and 95% CIs from the single logistic regressions of pAKI occurrence according to the serum syndecan-1 concentration at each time point. Serum syndecan-1 concentrations at T4 and T6 were associated with an increased risk of pAKI (T4: OR = 1.10 [1.01 to 1.21, *P* = 0.03]; T6: OR = 1.16 [1.01 to 1.34, *P* = 0.04]). To calculate the cutoff value for the occurrence of pAKI, ROC analysis was performed for the serum syndecan-1 concentrations at T4 and T6. The estimated cutoff value was 101.0 ng ml^−1^ (sensitivity: 0.71; specificity: 0.62; area under the curve (AUC) = 0.67 (0.51 to 0.83)) at T4 and 57.1 ng ml^−1^ (sensitivity: 0.82; specificity: 0.56; AUC = 0.71 (0.57 to 0.86)) at T6 (Fig. 4). To analyze whether this cutoff value was significantly associated with the development of AKI, a χ2 test was performed. The cutoff value at T4 was not significantly associated with the occurrence of pAKI (χ2 (1) = 0.34, *P* = 0.56), whereas the cutoff value at T6 was significantly associated with the occurrence of pAKI (χ2 (1) = 4.55, *P* = 0.03). Finally, to evaluate the association between the serum syndecan-1 concentration at T6 and the occurrence of pAKI, multivariable logistic regression was conducted to control for the potentially confounding effects that are thought to be associated with pAKI (age, CPB duration, preoperative estimated glomerular filtration rate (eGFR)) and the serum syndecan-1 concentration at T6. Table 3 shows the ORs and 95% CIs from logistic regressions of pAKI occurrence adjusted for patient and setting characteristics, as described above. A high serum syndecan-1 concentration at T6 was associated with the occurrence of pAKI (OR = 1.02 (1.00 to 1.03), *P* = 0.03).

**Table 2:** Single logistic regressions of pAKI incidence according to the serum syndecan-1 concentration

	OR	95%CI	*P* value
T1	1.18	(0.90 to 1.55)	0.24
T2	0.98	(0.92 to 1.05)	0.61
T3	0.98	(0.92 to 1.05)	0.60
T4	1.10	(1.01 to 1.21)	0.03
T5	1.02	(0.98 to 1.05)	0.36
T6	1.16	(1.01 to 1.34)	0.04

(T1) After induction of anesthesia, (T2) 1 h after initiation of CPB, (T3) 2 h after initiation of CPB, (T4) 30 min after weaning from CPB, (T5) 2 h after weaning from CPB, (T6) postoperative day 1

**Table 3:** Logistic regressions of pAKI occurrence adjusted for patient and setting characteristics

	OR	95% CI	*P* value
age	1.01	0.95 to 1.07	0.87
CPB duration	0.99	0.98 to 1.00	0.22
preoperative eGFR	0.93	0.88 to 0.99	0.03
syndecan-1 at T6	1.02	1.00 to 1.03	0.03

*CPB* Cardiopulmonary bypass, *eGFR* estimated glomerular filtration rate

## Discussion

In this study, we investigated the perioperative serum syndecan-1 concentration in patients who underwent CVS with CPB as an indicator of vascular endothelial damage and evaluated its impact on pAKI. The serum syndecan-1 concentration began to increase after the initiation of CPB and then exhibited a bimodal peak. Serum syndecan-1 concentrations at all time points were significantly elevated compared to those at T1. Simple linear regression analysis revealed that the serum syndecan-1 concentration at T4 and T6 influenced the occurrence of pAKI. The estimated cutoff values were 101.0 ng ml^−1^ and 57.1 ng ml^−1^, respectively. The cutoff value of the serum syndecan-1 concentration at T6 was also shown to independently influence pAKI occurrence according to the χ2 test. The results of logistic regression analysis adjusted for covariates thought to be associated with the occurrence of pAKI also indicated that the serum syndecan-1 concentration at T6 was associated with pAKI.

The glycocalyx is a complex consisting of a core protein that penetrates vascular endothelial cells, glycosaminoglycans, hyaluronic acid, and other sugar chains [1]. The vascular endothelial glycocalyx has various physiological functions, including regulating vascular permeability, regulating leukocyte migration, and inhibiting thrombosis [9, 10]. Syndecan-1 is a core protein of the glycocalyx. When the glycocalyx is disrupted and detached from the vascular endothelium, the blood concentration of syndecan-1 increases, indirectly providing an estimate of the degree of glycocalyx disruption [11].

Several studies have shown that various factors associated with CVS, such as sympathetic hyperactivity, ischemia–reperfusion injury, induction of inflammatory conditions, and volume overload, can cause vascular endothelial cell injury [12–14]. Bruegger et al. measured the intraoperative serum syndecan-1 concentration in patients who underwent on-pump or off-pump coronary artery bypass grafting (CABG). In the on-pump CABG group, the serum syndecan-1 concentration increased twofold above the baseline level at the time of aortic occlusion release and further increased at weaning from CPB. On the other hand, a significant increase in the serum syndecan-1 concentration was also observed in the off-pump CABG group at the end of central anastomosis [15]. Rehm et al. showed trends in the serum syndecan-1 concentration during ascending aortic replacement surgery involving CPB and abdominal aortic replacement surgery involving aortic occlusion. The serum syndecan-1 concentration increased by 65-fold above the preoperative level 2 min before weaning from CPB during ascending aortic replacement surgery and increased 15-fold above the preoperative level 15 min after the withdrawal of aortic occlusion during abdominal aortic replacement surgery [16]. He et al. measured the perioperative serum syndecan-1 concentration in patients who underwent CVS with CPB. The serum syndecan-1 concentration began to increase within 10 min after CPB initiation and peaked when the aortic occlusion was released [17]. In previous studies, the serum syndecan-1 concentration during CPB peaked at the time of weaning from CPB and then rapidly declined due to rapid metabolism from the kidneys [5]. In contrast, in the present study, the serum syndecan-1 concentration temporarily decreased at 30 min after weaning from CPB and then increased again. There are several possible reasons for this discrepancy. First, in the present study, the duration of CPB and aortic occlusion was longer than that in previous studies, and the longer duration of CPB and aortic occlusion may have resulted in greater damage to the vascular endothelium. Second, massive infusions have been reported to cause glycocalyx disorders by promoting the release of ANP [13, 14]. Although the present study had insufficient data on blood transfusions after weaning from CPB, excessive infusion may be associated with glycocalyx disorders.

In the present study, there was a significant difference in the serum syndecan-1 concentration between the pAKI group and non pAKI group at T6. Univariate analysis revealed that serum syndecan-1 concentrations at T4 and T6 were associated with pAKI. Several studies have investigated the relationship between vascular endothelial damage and pAKI in patients with CVS. Saqib H. Qureshi et al. used porcine models to compare a group undergoing surgery with CPB and a group undergoing sham surgery to examine the effects of CPB on renal dysfunction, and they analyzed the effects of CPB on renal vascular endothelial cells using immunofluorescence probing. In their study, there was a significant reduction in creatinine clearance in Porcinis patients who underwent surgery with CPB. In addition, immunofluorescence probing revealed a significant decrease in glycosaminoglycan, a component of the glycocalyx, in renal vascular endothelial cells from porcine patients who underwent surgery with CPB compared to those who underwent a sham operation [3]. Several studies have also been conducted on humans. De Melo Bezerra Cavalcante et al. reported an association between early postoperative serum syndecan-1 concentrations and pAKI in children undergoing CVS. Their results showed that a decrease in the serum syndecan-1 concentration within 2 h after surgery was associated with the occurrence of pAKI. They also reported that the cutoff value of the serum syndecan-1 concentration that predicts the development of severe AKI (KDIGO stage 2 or 3) was 66.4 ng mL^−1^ [4]. Kim et al. measured the serum syndecan-1 concentration at the induction of anesthesia and at weaning from CPB in patients undergoing valvular surgery and analyzed the association between the serum syndecan-1 concentration and pAKI incidence. They found that a serum syndecan-1 concentration > 90 ng ml^−1^ at induction of anesthesia was associated with an increased risk of pAKI [5]. While these studies have shown an association between preoperative or postoperative serum syndecan-1 concentrations and the occurrence of pAKI, little is known about the association between serum syndecan-1 concentrations measured throughout the perioperative period and the occurrence of pAKI. The present study differs from previous studies in that the perioperative serum syndecan-1 concentration was frequently measured to determine the trends and associations of these changes with the occurrence of pAKI.

This study has several limitations. First, since this was a small, single-center study, it is not clear whether this is true for all races, and further data validation in patients with the same background is needed to determine whether the cutoff values derived from this study are correct. Second, patients with preexisting renal dysfunction (serum creatinine level ≥ 2 mg dl^−1^) were excluded from the study. This may also explain why no difference in the baseline serum syndecan-1 concentration was found**.** However, whether the results of the present study apply to patients with chronic renal failure is unknown and requires further investigation. Third,

## Conclusion

The trends in the perioperative serum syndecan-1 concentration and its association with the occurrence of pAKI in patients undergoing CVS with CPB were investigated. The serum syndecan-1 concentration began to increase after the initiation of CPB and then exhibited a bimodal peak. The serum syndecan-1 concentration at all time points was significantly elevated compared to that after the induction of anesthesia. The serum syndecan-1 concentration on postoperative day 1 was associated with the occurrence of pAKI. The estimated cutoff value was 57.1 ng ml^−1^ (sensitivity = 0.82, specificity = 0.56, AUC = 0.71 (0.57 to 0.86)). Multivariate analysis revealed that the serum syndecan-1 concentration on postoperative day 1 was associated with the occurrence of pAKI.

## Data Availability

The datasets used and/or analyzed during the current study are available from the corresponding author upon reasonable request.

## Abbreviations

CVS: Cardiovascular surgery
CPB: Cardiopulmonary bypass
pAKI: Postoperative acute kidney injury
EuroSCOREII: European System for Cardiac Operative Risk Evaluation II
eGFR: Estimated glomerular filtration rate
LVEF: Left ventricular ejection fraction
RCC: Red cell concentrate
FFP: Flesh frozen plasma
